# The Inflammatory Phenotype in Failed Metal-On-Metal Hip Arthroplasty Correlates with Blood Metal Concentrations

**DOI:** 10.1371/journal.pone.0155121

**Published:** 2016-05-26

**Authors:** Erja-Leena Paukkeri, Riku Korhonen, Mari Hämäläinen, Marko Pesu, Antti Eskelinen, Teemu Moilanen, Eeva Moilanen

**Affiliations:** 1 The Immunopharmacology Research Group, University of Tampere School of Medicine and Tampere University Hospital, Tampere, Finland; 2 Immunoregulation, Institute of Biomedical Technology, University of Tampere, Tampere, Finland; 3 Department of Dermatology, Tampere University Hospital, Tampere, Finland; 4 Coxa Hospital for Joint Replacement, Tampere, Finland; Delft University of Technology (TUDelft), NETHERLANDS

## Abstract

**Introduction:**

Hip arthroplasty is the standard treatment of a painful hip destruction. The use of modern metal-on-metal (MOM) bearing surfaces gained popularity in total hip arthroplasties during the last decade. Recently, worrisome failures due to adverse reaction to metal debris (ARMD), including pseudotumor response, have been widely reported. However, the pathogenesis of this reaction remains poorly understood. The aim of the present study was to investigate the ARMD response by flow cytometry approach.

**Methods:**

Sixteen patients with a failed Articular Surface Replacement (ASR) hip prosthesis were included in the study. Samples of pseudotumor tissues collected during revision surgery were degraded by enzyme digestion and cells were typed by flow cytometry. Whole blood chromium and cobalt concentrations were analyzed with mass spectrometry before revision surgery.

**Results:**

Flow cytometry analysis showed that the peri-implant pseudotumor tissue expressed two principal phenotypes, namely macrophage-dominated and T-lymphocyte-dominated response; the average portions being 54% (macrophages) and 25% (T-lymphocytes) in macrophage-dominated inflammation and 20% (macrophages) and 54% (T-lymphocytes) in T-lymphocyte-dominated response. The percentages of B-lymphocytes and granulocytes were lower in both phenotypes. Interestingly, the levels of blood chromium and cobalt were significantly higher in patients with macrophage-dominated response.

**Conclusions:**

The results suggest that the adverse tissue reactions induced by MOM wear particles contain heterogeneous pathogeneses and that the metal levels are an important factor in the determination of the inflammatory phenotype. The present results support the hypothesis that higher metal levels cause cytotoxicity and tissue injury and macrophages are recruited to clear the necrotic debris. On the other hand, the adverse response developed in association with lower metal levels is T-lymphocyte-dominated and is likely to reflect hypersensitivity reaction.

## Introduction

Total hip arthroplasty (THA) is an established and well documented operative treatment of a painful hip destruction associated with osteoarthritis (OA) or rheumatoid arthritis (RA). Although the conventional hip arthroplasty has a good reputation, the factor limiting its durability especially in younger THA patients has been the wear of polyethylene (PE) socket [1,2]. Metal-on-metal articulation (MOM) has been used for decades as a more wear-resistant alternative to conventional metal-on-polyethylene bearing in THA [3]. The recent MOM hip boom was initiated by the encouraging early results obtained using hip resurfacing and THA implants [4,5]. Most major companies introduced their versions of the resurfacing implant, followed by adaptation of MOM bearing surfaces into conventional stemmed THA devices. Early clinical reports of these second generation MOM hips were promising, and the method was soon widely used: for example, in the US the proportion of MOM bearing surface was 35% of total hip arthroplasties in 2005–2006 [6].

Only after several years of routine clinical use of MOM hip implants, reports of failures started to emerge. It became apparent that in some patients the metal wear debris induces a local inflammatory reaction, now known as “adverse reaction to metal debris” (ARMD) [7–10]. Clinical features associated with ARMD and increased failures of MOM hips include suboptimal acetabular component positioning [11,12] leading to edge loading and excessive production of metal wear particles [13] but the reaction may occur also in perfectly well aligned implants. Also, the head size of the MOM implant seems to relate to the risk and the incidence of ARMD has been reported to be higher in females [12,14]. Further, there are clear differences between the MOM implant designs, the Articular Surface Replacement (ASR) being the one associated with most frequent ARMD reactions [12,15,16]. The poor performance of ASR prostheses in the British National Joint Registry prompted a medical device alert by the authorities, followed by a voluntary recall of the product by the manufacturer DePuy [17,18].

According to the studies analyzing the cases with a clinical failure of a MOM, ARMD is widely present regardless of the type of the implant. At the same time, the inflammatory process related to the ARMD reaction remains poorly understood in many aspects. Already in 1970s Jones and co-workers reported of fluid-filled capsules and necrotic tissue around metallic McKee devices [19] mimicking the pseudotumour like reaction seen also in association with the failure of many modern MOM devices. Histologically, the tissue displays an inflammatory appearance with cystic and solid masses with tissue debris and inflammatory cells [7,9,20–23]. Macrophages, many of which contain metal particles, are usually seen. Also lymphocytes are present and they often appear as aggregates around post-capillary venules. This type of histological appearance is often called aseptic lymphocyte-dominated vasculitis associated lesion (ALVAL) [7,20]. Prominent lymphocyte infiltration has given rise to a hypothesis that metal wear particles elicit a delayed-type hypersensitivity reaction [7]. However, the results of epicutaneous skin-tests performed to the patients with a failed MOM implant have been contradictory [24] and as described in recent reviews [23,25,26] hypotheses of foreign-body reaction and cytotoxicity have also been expressed, especially as ALVAL characteristics are not always found in pseudotumour tissue.

The histology of ARMD has been beautifully described [7,20–23,27]. In the present study, we aimed to have another approach to investigate and understand the cell types and mechanisms involved in ARMD by utilizing flow cytometry to further characterize the tissue reaction in failed MOM implants using tissue obtained from ASR revisions as an example.

## Patients and Methods

### Patients

The study was approved by the Ethics Committee of Tampere University Hospital, Tampere, Finland, and complies with the declaration of Helsinki. All patients provided their written informed consent.

Pseudotumor tissue from sixteen consecutive revisions of Articular Surface Replacement (ASR, DePuy, Warsaw, IN, USA) hip arthroplasties carried out at Coxa Hospital for Joint Replacement, Tampere, Finland were collected and analyzed. The clinical characteristics of the patients are described in Table 1. All primary operations had been performed for the treatment of end-stage osteoarthritis.

**Table 1:** Patient Characteristics.

Patient	Gender	Age (yr)	ASR implant type	ASR implant *in-situ* time (months)	Reason for revision
1	male	46	ASR XL modular	22	extremely high blood metal ion levels
2	female	49	ASR XL modular	31	extremely high blood metal ion levels
3	female	58	ASR XL modular	30	high blood metal ion levels, pseudotumour
4	male	75	ASR XL modular	39	high blood metal ion levels, pseudotumour, symptoms
5	male	65	ASR XL modular	42	high blood metal ion levels, pseudotumour, symptoms
6	female	54	ASR resurfacing	67	symptoms
7	female	46	ASR XL modular	81	high blood metal ion levels, symptoms
8	female	73	ASR resurfacing	63	high blood metal ion levels, pseudotumour, symptoms
9	male	74	ASR XL modular	43	high blood metal ion levels, pseudotumour, symptoms
10	female	64	ASR resurfacing	85	high blood metal ion levels, pseudotumour
11	female	56	ASR resurfacing	29	high blood metal ion levels, pseudotumour, symptoms
12	male	52	ASR XL modular	62	high blood metal ion levels, symptoms
13	female	63	ASR XL modular	52	moderately elevated blood metal ion levels, symptoms
14	male	67	ASR XL modular	54	high blood metal ion levels, pseudotumour, symptoms
15	female	64	ASR XL modular	76	high blood metal ion levels
16	male	65	ASR XL modular	39	high blood metal ion levels, pseudotumour, symptoms

### Reasons for Revision Surgery

Revision surgery of a MOM hip was considered if 1) a thick-walled pseudotumour with atypical contents or a solid pseudotumour was seen in cross-sectional imaging regardless of symptoms and whole blood metal ion levels; or 2) the patient had both elevated metal ion levels and hip symptoms despite a normal finding on cross-sectional imaging; or 3) increasingly and significantly symptomatic hip regardless of imaging findings or metal ion levels [28]. Symptoms included hip pain, discomfort, sense of instability, and/or impaired function of the hip as well as sounds from the hip. Infection was ruled out by at least five bacterial cultures obtained during revision surgery.

### Cell Isolation

Peri-implant tissue was obtained directly from surgery; the necrotic mass was removed and the tissue was minced and digested fresh with a cocktail of 1 mg/ml of Liberase TM Research Grade (Roche, Mannheim, Germany) and 0.04/0.32 U/ml of Collagenase/Dispase (Roche, Mannheim, Germany) in RPMI 1640 (Lonza Group Ltd, Basel, Switzerland) supplemented with penicillin (100 U/ml), streptomycin (100 μg/ml) and amphotericin B (250 ng/ml) (all from Invitrogen/Life Technologies, Carlsbad, CA, USA) at 37°C for 2 hours. After the digestion, the cell suspension was passaged through a cell strainer, washed three times with cold phosphate buffered saline (PBS) containing 5% of heat-inactivated foetal bovine serum (FBS) (Lonza Group Ltd, Basel, Switzerland) and resuspended in PBS supplemented with 5% of FBS for flow cytometry analysis, or in RPMI 1640 supplemented with 10% of FBS and antibiotics (see above) for cell culturing.

### Cell Characterization by Flow Cytometry

1 x 10^6^ freshly isolated pseudotumor tissue -derived cells were first incubated with purified human Fc Receptor Binding Inhibitor (eBiosciences, San Diego, CA, USA) for 5 minutes and then stained with a combination of antibodies against the pan-leukocyte marker CD45 (labelled with fluorescein isothiocyanate, FITC; Becton, Dickinson and Company, Franklin Lakes, NJ, USA), macrophage marker CD14 (labelled with phycoerythrin Cy7, PE-Cy7; eBiosciences), T-lymphocyte marker CD3 (labelled with allophycocyanin Cy7, APC-Cy7; Becton, Dickinson and Company), B-lymphocyte marker CD19 (labelled with allophycocyanin, APC; eBiosciences) and pan-granulocyte marker CD15 (labelled with phycoerythrin, PE; Becton, Dickinson and Company) for 20 minutes. After the incubation, the cells were washed twice with PBS supplemented with 1% of FBS, fixed with 1% of paraformaldehyde for 10 minutes and again washed twice with PBS/1% FBS. The samples were examined by FACSCanto II flow cytometry (Becton, Dickinson and Company) and the data was analyzed using FlowJo software (Tree Star, Inc, Ashland, OR, USA). In the preliminary experiments both anti-CD14 (see above) and anti-CD68 (labelled with fluorescein isothiocyanate, FITC; eBiosciences) were used as macrophage markers; as the results were similar anti-CD14 was chosen for the further experiments.

### Cell Culture

Pseudotumor tissue -derived cells were cultured (in two duplicates per patient) in the density of 5 x 10^5^ cells/ml in 6-well plates in RPMI 1640 supplemented with 10% of FBS and antibiotics (see above) at 37°C in humidified 5% CO_2_ atmosphere. After 42 hours of incubation, the culture media were collected, the samples were centrifuged at 400 g for 10 minutes and the supernatants were stored at -20°C until assayed for IL-6 and TNFα.

### Enzyme-Linked Immunosorbent Assay (ELISA)

The concentrations of IL-6 and TNFα in the culture media were determined by ELISA using reagents from R&D Systems Europe, Abingdon, UK and the assays were run in duplicate.

### Metal Ion Concentrations

Whole blood samples were collected using twenty-one-gauge needle connected to a Vacutainer TM system (Becton, Dickinson and Company) and trace element blood tubes containing sodium ethylenediaminetetraacetic acid. In the Finnish Institute for Occupational Health (FIOH), standard operating procedures were established for Co and Cr measurement using dynamic reaction cell inductively coupled plasma (quadripole) mass spectrometry (Agilent 7500 cx, Agilent Technologies, Santa Clara, CA, USA). The FIOH laboratory is accredited and has been used in several published studies utilizing blood metal ion measurements [28–30].

### Statistics

Results of normally distributed data are expressed as mean ± standard error of mean (SEM) and non-normally distributed data as median ± quartile range and range of values as indicated. Statistical significance of the differences between groups was calculated either by unpaired t-test (with Welch correction when needed) or by Mann-Whitney test where appropriate. Differences were considered significant at * = *p*<0.05, ** = *p*<0.01, and *** = p<0.001.

## Results

### The Distribution of the Inflammatory Cells were Polarized either to Macrophages or T-Lymphocytes

Inflammatory cells present in the pseudotumour tissue were characterized by flow cytometry. CD45 antigen was used as a general hematopoietic cell marker. CD14 (confirmed with similar results with CD68) was used as a marker of macrophages and CD3 and CD19 antigens were used to identify T-lymphocytes and B-lymphocytes, respectively. CD15 was used as a pan-granulocyte marker.

Macrophages and T-lymphocytes were the most dominant subsets of CD45^+^ cells (Table 2). B-lymphocytes were less frequent than T-lymphocytes, but in four cases the proportion of B-lymphocytes was more than 10% of all leukocytes, 19.2% being the highest proportion (Table 2). The proportion of granulocytes was generally low (median 1.78%) and only in one case it exceeded 5% (Table 2).

**Table 2:** Inflammatory Cell Distributions and Whole Blood Metal Levels.

	Proportions of haematopoietic cells (%)					
Patient	Macro-phages	T cells	B cells	granulo-cytes	B-Cr (μg/l)	B-Co (μg/l)
1	45.7	27.0	no data	3.03	28.8	64.9
2	51.2	20.7	no data	2.24	36.2	114.3
3	42.7	42.4	0.09	4.01	13.3	14.2
4	30.4	50.2	1.20	2.06	3.5	13.8
5	53.7	29.9	7.25	1.15	20.6	18.7
6	15.0	48.5	8.92	4.90	2.9	2.5
7	30.4	31.8	19.2	1.44	12.2	20.0
8	56.8	15.8	0.05	13.0	45.2	113.3
9	68.4	18.5	0.34	3.54	9.3	22.1
10	43.9	34.9	12.3	0.73	53.5	96.9
11	55.2	27.0	3.07	1.13	11.8	14.2
12	15.7	54.8	6.44	1.00	8.2	13.2
13	16.3	59.3	0.73	1.17	1.3	5.9
14	25.8	54.2	11.5	1.50	2.7	10.7
15	29.4	31.8	0.72	2.15	21.6	36.3
16	18.1	55.1	12.3	0.98	3.3	10.5

B-Cr = whole blood chromium; B-Co = whole blood cobalt

CD45^+^ cells were defined as hematopoietic cells, CD45^+^CD14^+^ as macrophages, CD45^+^CD3^+^ as T-lymphocytes (T cells), CD45^+^CD19^+^ as B-lymphocytes (B cells) and CD45^+^CD15^+^ as granulocytes.

**Fig 1:**
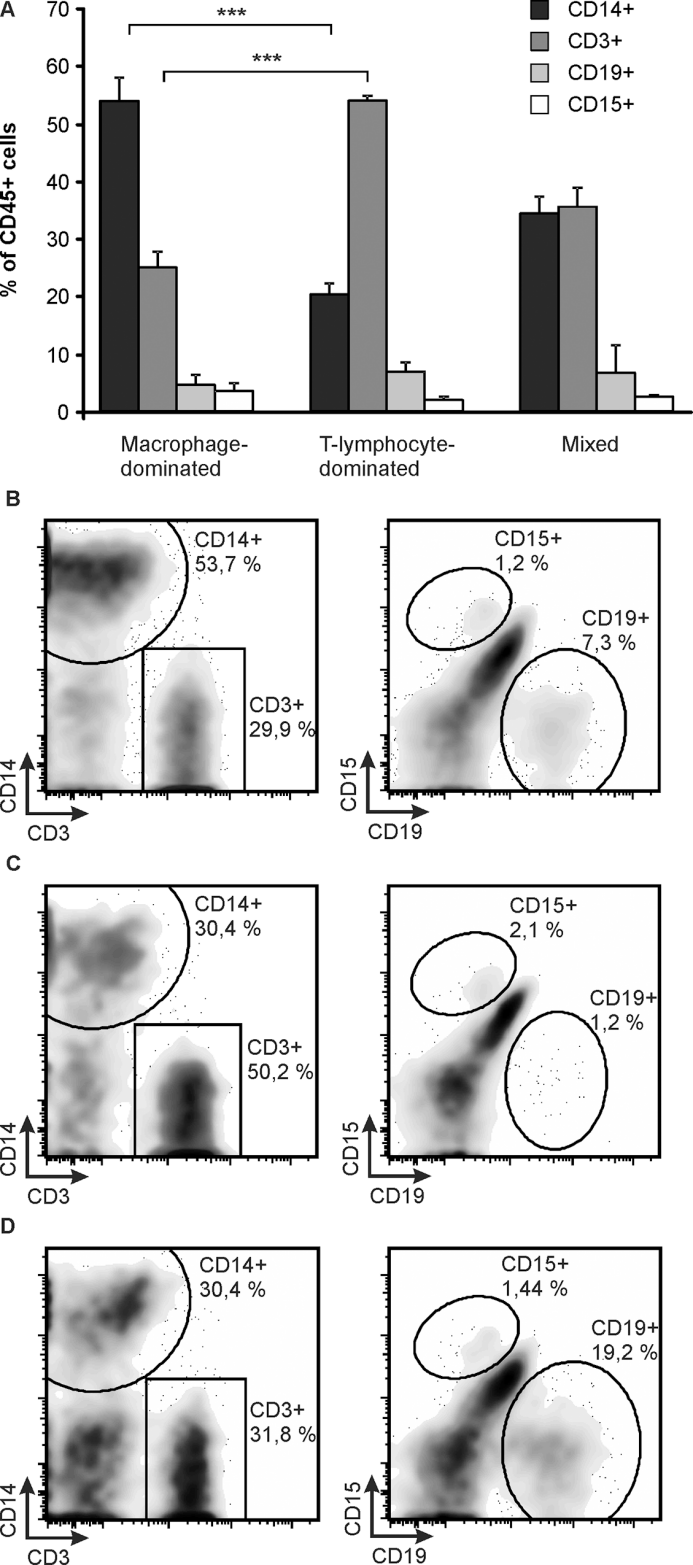
Flow Cytometry Analysis of CD45^+^ Cells Derived from Pseudotumor Tissue. CD45^+^ cells were gated and proportions of CD14^+^, CD3^+^, CD15^+^ and CD19^+^ cells were analyzed. The cases were divided into three groups according to CD14^+^ and CD3^+^ cell proportions, namely macrophage-dominated (B), T-lymphocyte-dominated (C) and mixed (D) groups. Average values (mean + SEM) of the proportions of different cell types in the three different phenotypes of inflammation (A) with representative flow cytometry blots (B-D) are shown. n = 7 in macrophage-dominated group, n = 6 in T-lymphocyte-dominated group and n = 3 in mixed group. ***p<0.001

Interestingly, most of the samples were clearly dividable into two groups according to their cell distributions (Fig 1). Seven of the 16 samples displayed macrophage-dominated cell distribution and six of the 16 were T-lymphocyte-dominated. In the macrophage-dominated group, the average (mean ± SEM) proportion of macrophages was 53.5 ± 3.0% and that of T-lymphocytes 24.8 ± 2.5%. While the corresponding values were 20.2 ± 2.5% (macrophages) and 53.6 ± 1.5% (T-lymphocytes) in the T-lymphocyte-dominated group. There were no overlapping cases in the proportions of either macrophages or T-lymphocytes between the two groups. The remaining three samples were classified as mixed reaction as they had about equal percentage of CD14 and CD3 positive cells. The three groups had comparable percentages of B-lymphocytes and granulocytes (Fig 1).

### IL-6 and TNFα Production was Elevated in Macrophage-Dominated Response

**Fig 2:**
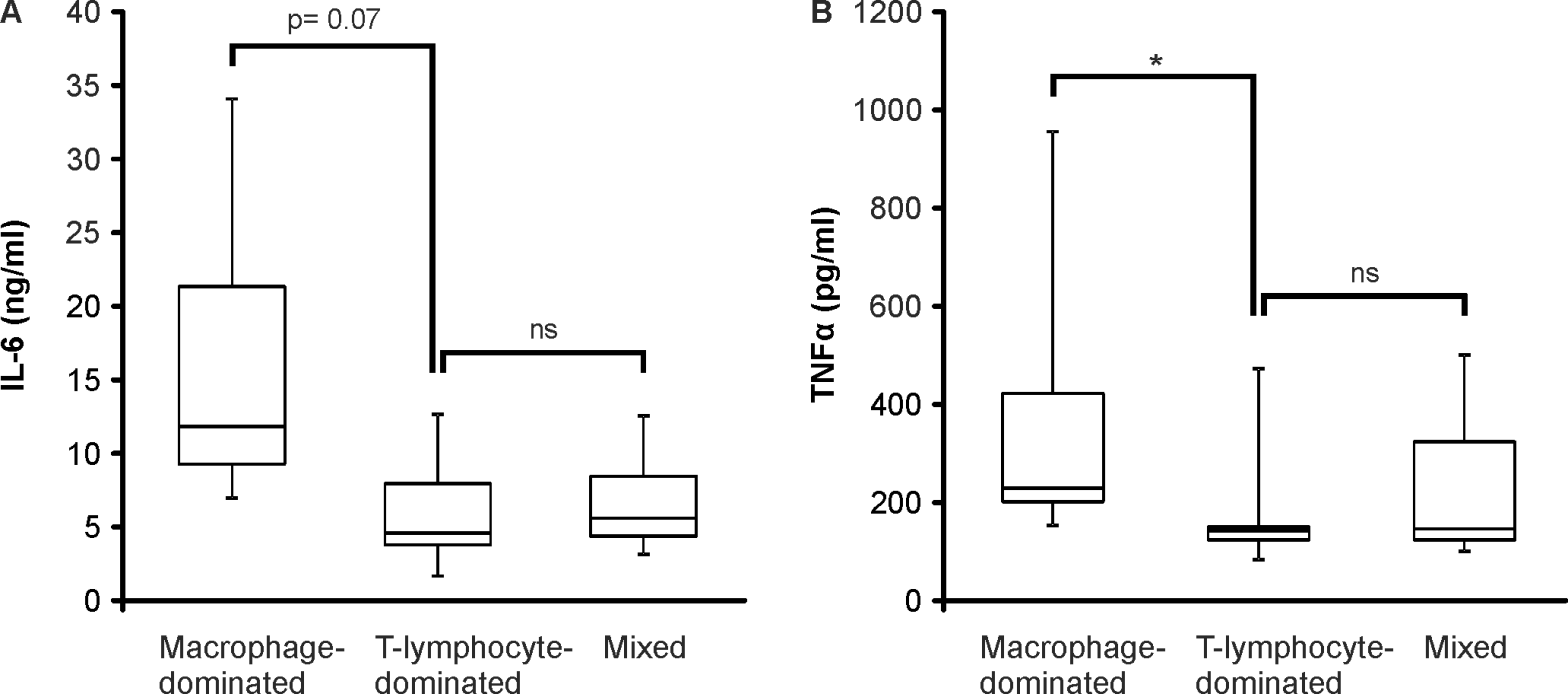
The Production of IL-6 and TNFα by Cells Derived from Pseudotumor Tissue. Samples of pseudotumor tissue were degraded by enzyme digestion and cell suspensions were cultured for 42 hours. Thereafter IL-6 (A) and TNFα (B) accumulated into the culture medium were measured by ELISA. Boxplots represent medians and interquartile ranges and whiskers indicate range of values. n = 7 in macrophage-dominated group, n = 5 in T-lymphocyte-dominated group and n = 3 in mixed group. *p<0.05.

Next, we analyzed the IL-6 and TNFα production of the cells derived from pseudotumor tissue. The cells were cultured for 42 hours and the levels of IL-6 and TNFα were measured in the culture medium by ELISA. TNFα production was higher in the macrophage-dominated group than in the T-lymphocyte-dominated group (p<0.05) and IL-6 production showed a similar trend (p = 0.07) as seen in Fig 2.

### Blood Metal Ion Concentrations were Related to the Inflammatory Cell Distribution in the Pseudotumor Tissue

All of the patients had increased blood metal ion levels with a significant variation between the patients. The metal ion levels of either blood chromium or cobalt exceeded the action level of 7 ppb proposed in the Medicines and Healthcare Products Regulatory Agency (MHRA, UK) guidelines [31] in 14 out of 16 patients. The median (range) of blood chromium concentration was 12.0 (1.3–53.5) μg/l and the median of blood cobalt level was 16.5 (2.5–114.3) μg/l (Table 2).

**Fig 3:**
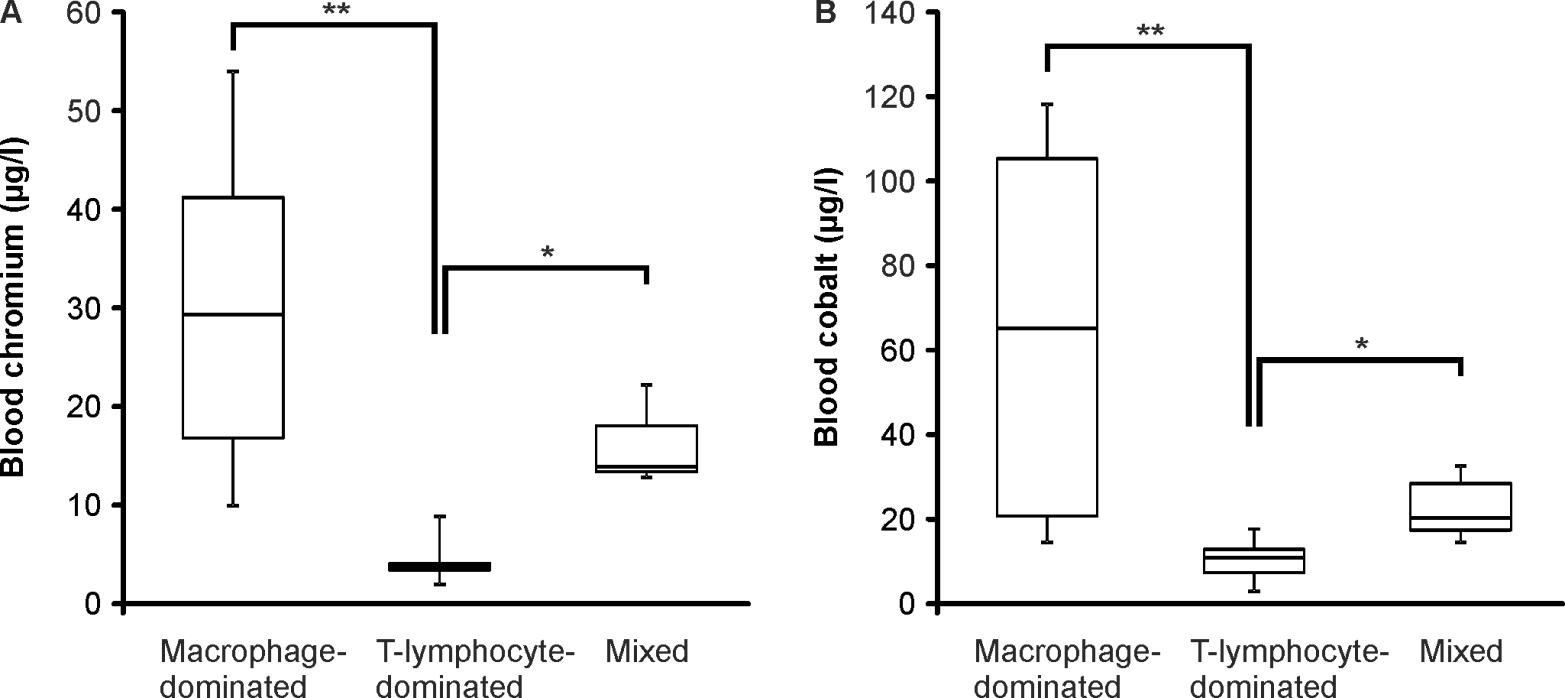
The Blood Chromium and Cobalt Levels. Whole blood samples were taken preoperatively from the patients and chromium (A) and cobalt (B) concentrations were analyzed by inductively coupled plasma mass spectrometry. Boxplots represent medians and interquartile ranges and whiskers indicate range of values. n = 7 in macrophage-dominated group, n = 5 in T-lymphocyte-dominated group and n = 3 in mixed group. *p<0.05, **p<0.01

When the levels of blood chromium and cobalt in patients with macrophage or T-lymphocyte -dominated inflammatory phenotype were compared, a remarkable difference between the groups was found. Both the chromium and cobalt levels were significantly higher in patients with macrophage-dominated inflammation than in the patients with T-lymphocyte-dominated response with the median levels being 28.8 μg/l (Cr) and 64.9 μg/l (Co) in the macrophage-dominated group and 3.1 μg/l (Cr) and 10.6 μg/l (Co) in the T-lymphocyte-dominated group (Fig 3). The small group of patients with similar macrophage and T-lymphocyte abundance had median blood chromium level of 13.3 μg/l and cobalt level of 20.0 μg/l.

## Discussion

The adverse tissue reactions related to MOM implants are presently a major topic in hip arthroplasty debates, but the understanding on the detailed cellular mechanisms eventually resulting in the development of the reaction remains limited and various hypotheses have been presented [7,20,23–27]. The present study shows that the inflammatory activation related to failed ASR implants is heterogeneous and can be characterized either as s macrophage or as a T-lymphocyte dominated reaction. Interestingly, the two types of responses differed also according to the blood metal ion concentrations in a manner that the macrophage-dominated phenotype was associated with higher blood levels of chromium and cobalt.

The histology of ARMD has been described in a number of publications, e.g. [7,20–23,27]. The present study extends the previous data by utilizing flow cytometry analysis in the characterization of cell types in the pseudotumor tissue of revised MOM hips. The pseudotumour tissue was degraded by enzyme digestion and the resulting cell suspension was analyzed by flow cytometry. Therefore, results on the cell typing are more representative for the entire pseudotumor tissue reaction than the usual histology which is limited to the sections investigated. Further, the flow cytometric data can be analyzed in a more quantitative manner.

Our results show that the leukocyte populations in the ARMD are dominated either by macrophages or by T-lymphocytes but the percentage of the polymorphonuclear leukocytes (i.e. CD15 positive cells including both neutrophils and eosinophils) remained rather low, the median being less than 2% of all leukocytes. Eosinophils have sometimes been noted in the histological studies [7,9] but in the present study we did not specify the granulocyte subtypes. However, the low percentage of granulocytes suggests that neither direct eosinophil mediated allergic response nor neutrophil mediated innate immune response plays any major role in the pathogenesis of the ARMD in most patients. Further, infection was ruled out with multiple bacterial cultures of samples collected during the revision surgery. Intriguingly, also the percentage of B-lymphocytes was lower than that of T-lymphocytes in all patients supporting a major role of the latter in the lymphocyte mediated responses. However, in four patients the proportion of B-lymphocytes exceeded 10% of all leukocytes. Based on a recent study [27], also B-lymphocytes may have a significant role in the pathogenesis of ARMD, at least in some patients as discussed below.

The present results show that macrophage-dominated ARMD was typical for those patients who had higher blood metal ion concentrations. This suggests that the metal debris induces cytotoxicity or other phenomena that attract macrophages to the pseudotumor tissue. Cobalt-chromium alloys are especially hard, but some amounts of chromium and cobalt particles or soluble metal are released from hip devices by the wear or corrosion of the implant [32]. This is reflected as increased chromium and cobalt concentrations in the circulation [28–30,33,34] as also detected in the present study. In contrast to the micrometer-sized wear debris particles produced by polyethylene bearings, the wear particles of MOM articulations have been found to be primarily less than 50 nm in size [35,36]. The physiochemical and biological corrosion also results in the release of soluble metal ions, from the larger particles and from the devices, and the blood metal concentrations depict the concentrations of soluble metal ions surrounding the MOM device [37].

Activated macrophages phagocytize wear particles, which in the case of ARMD is evidenced by metal particles within the cells [23,26,32]. Cobalt and chromium ions can also be transported into different cell types via non-specific anion carriers. Inside the cells, both cobalt and chromium can induce the formation of reactive oxygen species, DNA and chromosome damage, and cytotoxicity. [32,38] *In vitro* studies show that cobalt is more toxic than chromium: cobalt nanoparticles have been reported to induce direct cell death at concentrations of 1 x 10^12^ particles/ml and cobalt ions at concentrations of 1000 μM [39]. Based on electron microscopic examinations, cobalt cytotoxicity is associated with damage in mitochondria and cellular membranes as well as disruption of cellular metabolism [39,40].

Furthermore, cobalt is known to induce cellular changes mimicking hypoxia, mainly through enhancing the levels of transcription factor hypoxia inducible factor 1 alpha (HIF-1α) [41,42]. Therefore it is likely that cobalt ions released from MOM devices induce inflammatory reactions and cytotoxicity typical for hypoxia. In support, it was recently reported based on immunohistochemical staining that HIF-1α levels were increased in the peri-implant tissue of failed MOM implants as compared to that of failed MOP implants [43]. Further, enhanced HIF-1α expression was associated with increased production of reactive oxygen species and the inflammatory cytokine TNFα. Cobalt ions may also have a direct stimulatory effect on macrophages. Tyson-Capper and co-workers [44] reported recently, by using reporter cell line, that cobalt ions directly activated toll-like receptor 4 (TLR4). TLR4 is widely expressed in macrophages and has a significant role in triggering innate immune responses following exposure to e.g. lipopolysaccharide and other bacterial products.

The data described above give a potential explanation to our present finding that high blood cobalt and chromium concentrations are associated with macrophage-dominated inflammatory response in the pseudotumor tissue around failed MOM hips. Taken together, we hypothesize that high blood metal levels reflect high peri-implant tissue levels of nanometer-sized metal particles and metal ions, especially cobalt, which cause tissue injury and cell necrosis around MOM hips. The damaged tissue recruits macrophages, which are the professional cells to phagocytize necrotic cells and clean the tissue debris. This assumption is supported by the histological finding that macrophages in the pseudotumor tissue contain small droplike inclusions which resemble phagocytized organic material but not implant-derived wear debris [7]. In addition, the macrophages infiltrated into the peri-implant tissue are further activated by the metal nanoparticles and soluble metal ions released locally from the MOM devices. That further magnifies the inflammatory reaction in the pseudotumor tissue, e.g. through increased production of proinflammatory cytokines IL-6 and TNFα as shown in the present study. This sequence of events and the cytotoxic mechanisms of the metal debris and released metal ions described above are proposed to form the basis of the pathogenesis of the macrophage-dominated type of ARMD found in the present study.

A perivascular lymphocyte reaction is another characteristic feature in ARMD response [7,20,23]. Our results suggest that the response is T-cell-dominated; and in about half of the cases the T-lymphocyte accumulation is a predominant feature over the macrophage infiltration. According to the hypothesis of delayed hypersensitivity reaction, the metal ions released from the implant can form complexes with tissue proteins and the aberrant metal-protein complexes may be recognized by the immune system as foreign antigens and result in the activation of the adaptive immune system. Lymphocyte infiltration into pseudotumor tissue supports this hypothesis, but the results of hypersensitivity skin-tests performed to the patients with failure of MOM implant have been contradictory [19,24,45–47]. However, it is challenging to establish the connection between peri-implant tissue reaction and hypersensitivity measured by skin tests since the immunological reactions evoked by transient cutaneous exposure to a pre-determined allergen may differ from that induced by the constant exposure to an orthopaedic implant which may give rise to development of uncharacterized metal-modified endogenous molecules [24,48]. Other methods to measure hypersensitivity, like lymphocyte transformation test or migration test have still been only in a minor use [24]. In our study, when an inflammatory pseudotumor reaction developed in patients who had only slightly increased whole blood metal concentrations, the response showed T-lymphocyte-dominated phenotype. That points primarily to metal-induced hypersensitivity reaction as the pathogenesis of the inflammatory reaction around the hip arthroplasties rather than to metal-induced cytotoxicity.

In histological studies of ARMD, lymphocyte aggregates containing B-lymphocytes in addition to T-cells have been detected in a subpopulation of patients [7,27]; and this is supported by the present finding that a quarter of our patients had moderate levels of B-lymphocytes. Furthermore, the T and B-lymphocyte containing lymphoid aggregates were reported to display features typical for tertiary lymphoid organs [27]. This finding suggests that also autoimmunity to metal-carrier complexes may be involved in the pathogenesis of lymphocyte-dominated ARMD [23,27].

After all, we hypothesize that high local metal concentrations in the pseudotumor tissue result in cellular necrosis which recruits macrophages into the tissue to perform their basic immunological function, i.e. to phagocytize necrotic cells and clean tissue debris. However, if ARMD is developed in a situation where metal levels are only moderately elevated, delayed hypersensitivity reaction characterized by dense accumulation of T-lymphocytes is a more likely pathophysiological mechanisms. Metal sensitivity due to previous exposure e.g. to metal containing medical devices or metal necklace is not uncommon in the population. In addition, susceptible individuals can develop delayed hypersensitivity reaction to altered metal-conjugated host proteins formed in the presence of MOM-released ions or nanoparticles. Also, autoimmune mechanisms may contribute to the lymphocyte-mediated response. However, the proposed hypothesis on the dominant role of the metal concentrations in the inflammatory phenotype of ARMD needs to be further investigated since on the basis of our results, it is not possible to conclude if these two inflammatory conditions are distinct from each other or if they are interactive or consecutive processes. Also, the number of patients in our study was relatively small.

In conclusion, the present results show that there are distinct inflammatory phenotypes characterizing the adverse reaction to metal debris in patients with a failed ASR metal-on-metal hip which may reflect different disease mechanisms. The macrophage-dominated response was associated with higher blood metal ion concentrations and cytokines typical for innate immune response. This is proposed to depict metal-induced cytotoxicity resulting in massive macrophage infiltration and macrophage-mediated clearance of the necrotic tissue. While ARMD reaction associated with lower metal concentrations was characterized by T-lymphocyte-dominated tissue response applicable to hypersensitivity response.
